# Diagnostic validity of optic nerve head colorimetric assessment and optical coherence tomography angiography in patients with glaucoma

**DOI:** 10.1136/bjophthalmol-2020-316455

**Published:** 2020-07-23

**Authors:** Carmen Mendez-Hernandez, Surina Wang, Paula Arribas-Pardo, Liseth Salazar-Quiñones, Noemi Güemes-Villahoz, Cristina Fernandez-Perez, Julian Garcia-Feijoo

**Affiliations:** 1 Ophthalmology, Hospital Universitario Clinico San Carlos, Madrid, Spain; 2 Inmunología, Oftalmología y ORL, Facultad de Medicina., Universidad Complutense, Madrid, Spain; 3 OFTARED, Madrid, Spain; 4 Ophthalmology, Hospital Central de la Defensa Gomez Ulla, Madrid, Spain; 5 Medicina Preventiva, Complejo Hospitalario Universitario de Santiago de Compostela, Santiago de Compostela, Spain

**Keywords:** Glaucoma, Imaging, Optic Nerve, Macula

## Abstract

**Background/Aims:**

The aim of this study was to assess the optic nerve head (ONH) and macular vessel density with optical coherence tomography angiography (OCT-A) and the ONH haemoglobin (ONH Hb) amount with Laguna ONhE program in open-angle glaucoma (OAG) patients.

**Methods:**

In this prospective observational cross-sectional study, 67 OAG patients and 41 healthy age-sex frequency matched subjects were examined with OCT-A and retinal photos. The circumpapillary (wcpVD), optic nerve head (iVD) and macular (wmVD) capillary vessel density of OCT-A and ONH colorimetric assessment to determine the ONH Hb amount using the Laguna ONhE program were evaluated.

**Results:**

Significant differences between normal subjects and glaucoma patients in the wcpVD (22.18±3.42 vs 16.03±2.89%; p<0.001), iVD (18.31±5.56 vs 12.52±4.67%; p<0.001), wmVD (15.60±2.34 vs 13.34±2.32%; p<0.001) and amount of ONH Hb (71.10±1.67 vs 68.86±2.46%; p<0.001) and in the papillary cup (68.14±5.25 vs 64.77±5.08%; p=0.001) were found. The Laguna ONhE glaucoma discriminant function (GDF) index had a negative value in the OAG patients and normal values in healthy subjects (−18.76±13.31 vs 7.98±14.09; p<0.001). The area under the receiver operating characteristic (ROC) curve (AUROC) for discriminating between healthy and glaucomatous eyes was highest for wcpVD (0.93; 95% CI 0.86 to 0.97, p<0.0001), followed by GDF (0.92; 95% CI 0.86 to 0.97, p<0.0001), iVD (0.79; 95% CI 0.70 to 0.86; p<0.0001) and ONH Hb (0.78; 95% CI 0.69 to 0.85, p<0.0001). Pair wise comparisons showed that the AUROC of wcpVD (0.93) was not significantly different than GDF (0.92) (p=0.855).

**Conclusion:**

Laguna ONhE program and OCT-A have similar diagnostic validity in open-angle glaucoma patients.

## INTRODUCTION

As previously demonstrated, intraocular pressure (IOP) increase is not the only responsible for the development and progression of glaucoma, although it is true that it is the most relevant and the only one on which effective therapeutic action can be carried out. Although large clinical trials conducted on glaucoma have not shown that vascular diseases imply a greater risk of glaucoma,^1–^ ^3^ dysregulation or vascular alteration in the development of the disease have been studied with greater intensity in recent years^4–^ ^6^ since some glaucoma patients show signs of a decrease in blood supply to the optic nerve head (ONH).^7 8^ The physiopathology of glaucoma is not well known yet but there is an increasing evidence that the vascular system, and specifically retinal microvasculature, plays an important role in the development of the disease.^9–^ ^11^ One of the technological advances that has contributed to the increased interest in the influence of the vascular factor on the development of glaucoma is optical coherence tomography with angiography or optical coherence angiotomography (OCT-A). This non-invasive technique obtains in vivo images of the microvascularisation of the papilla and the retina in a reproducible way using the principle of ‘movement contrast’ for the detection of blood flow by performing multiple sections of B-scans taken in the same section of the retina in a very short interval of time. The device identifies the signal that corresponds to red blood cells flow within the blood vessels. The OCT-A allows a separate assessment of deep and superficial microvasculature.^12^ With this non-invasive tool, circumpapillary, papillar and macular vessel density have been shown to be lower in glaucoma patients.^13 14^

**Figure 1:**
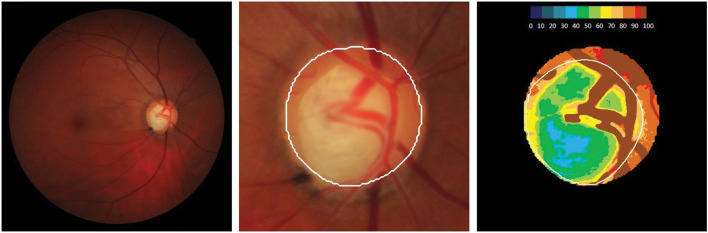
Retinography in a patient with glaucoma. The left image shows the colour fundus photograph of the optic nerve head. The right image shows the corresponding pseudo-image representing the amount of haemoglobin. Cold colours represent the areas of the head of the optic nerve with less haemoglobin. Warm colours show the parts of the papilla with the best perfusion.

Laguna ONhE program determines optic nerve head haemoglobin (ONH Hb) on retinal photographs based on detecting colour differences (figure 1). Laguna ONhE program has been previously described.^15^ It uses mathematical algorithms for automatic component segmentation in order to identify central retinal vessels. Thus, two areas of the ONH are defined: central retinal vessels and ONH tissue itself. The program analyse three components of ONH photographs: blue (B), green (G) and red (R) and applies the formula (R-G)/R to the pixels of vessels and tissue. The result obtained for the vessels is used as the reference value for calculating the haemoglobin content in the tissue. The (R-G)/R value is calculated for any area of the tissue, then divided by the (R-G)/R value for the vessels and the result is multiplied by 100. Thus, a relative measure (percentage) of the amount of haemoglobin in the tissue is obtained. In addition, the procedure can estimate the vertical cup/disc ratio as well as delimit the cup and rim, taking into consideration the amount of haemoglobin in the different papillary sectors. Its reproducibility, as well as its diagnostic capacity in glaucoma, have been demonstrated in previous studies, in which it has been compared with other structural diagnostic procedures already commercialised such as confocal tomography or spectral-domain optical coherence tomography (SD-OCT).^15–^ ^20^ However, no studies have been performed in order to evaluate its capacity of quantifying changes in glaucoma compared with OCT angiography. The aim of this study was to compare the diagnostic ability of both methods, taking into account visual field parameters, in patients with open-angle glaucoma.

## MATERIAL AND METHODS

### Subjects

In this prospective observational cross-sectional unicenter study, 67 OAG patients from our reference university hospital and 41 healthy age-sex frequency-matched subjects were included. The study protocol was approved by the Institutional Review Board of our Hospital, and followed the tenets of the Declaration of Helsinki. Informed consent was obtained from each participant before inclusion in the study.

As control subjects, healthy individuals were enrolled from the Ophthalmology Department of our hospital undergoing a routine test or OAG patients’ relatives (husband or wife).

Patients who met the eligibility criteria were prospectively recruited

Inclusion criteria were age greater than 18 years old, visual acuity of 20/40 or better, sphere between ±5 diopters cylinder between ±3 diopters and open-angle on gonioscopy. Only one eye randomly selected per subject was included in the final analysis.

Specific inclusion criteria for the glaucoma group were primary open-angle glaucoma history, repeatable glaucomatous perimetric damage or documented glaucoma damage at the optic disc.

Specific inclusion criteria for healthy subjects were IOP <21 mm Hg, normal aspect of the optic disc and no history of elevated IOP.

Eyes recently undergoing cataract surgery (<1 year) were excluded

Patients with other types of glaucoma such as normal tension glaucoma, angle-closure or neovascular glaucoma, as well as non-glaucomatous optic neuropathies, neuroophthalmological or retinal disease, neurological diseases such as dementia, arterial hypotension history to exclude patients with normotensive glaucoma risk factors, or any condition that could limit the ability to perform visual fields, were not included.

In order to report systemic disorders patients were asked which medication was being taken using a standardised questionnaire in which the following questions were included: have you been diagnosed with high blood pressure, diabetes or hypercholesterolaemia? If yes, when were you diagnosed and what medication are you currently taking? Women using post-menopausal hormone treatments were excluded to reduce the potential effect of oestrogen on vessel density values.

The outcome variable was the presence or absence of glaucoma. Glaucoma was defined as the presence of repeatable abnormal standard automated perimetry (SAP) results, focal (localised notching) or diffuse neuroretinal rim narrowing, concentric enlargement of the optic cup, or both.

An abnormal SAP was defined as reproducible glaucomatous visual field loss in the absence of any other abnormalities to explain the defect with mean defect (MD) greater than 2 dB or depressed points in the defect curve and loss variance higher than 7 dB^2^ using Octopus TOP G1 program, Octopus 600, Haag-Streit AG, Bern, Switzerland.

Parameters of each procedure were independently, blindly and prospectively measured

The independent variables analysed were glaucoma discriminant function (GDF) and the percentage of haemoglobin at the ONH using Laguna ONhE program and the circumpapillary vessel density of the whole image (wcpVD), vessel density at the optic nerve head (iVD) and macular capillary density of the whole image (wmVD) of OCT-A measured as percentage.

Age, gender, IOP measurement, MD, disc area (mm^2^), previous antiglaucomatous surgeries and number of ocular hypotensive treatments used were also recorded.

All participants underwent an ophthalmological examination, including visual acuity, refraction, slit-lamp biomicroscopy, IOP measurement with Goldmann applanation tonometry, gonioscopy, ultrasound pachymetry and dilated fundus examination. Perimetry was done and analysed for glaucoma patients, not for healthy subjects. Only one eye randomly selected per subject was included in the final analysis.

### Optical coherence tomography angiography

All participants were examined with the Nidek’s RS-3000 Advance OCT (Nidek, Gamagori, Japan), which automatically calculates peripapillary and macular vessel densities, to collect SD-OCT and OCT-A images. All the scans were obtained by the same operator without pupil dilation in a darkroom. The scanning was repeated if the SSI quality was <7/10. Poor OCT image quality due to segmentation errors secondary to extensive peripapillary changes that make OCT-A unreadable was not included.

The OCT-A RS-3000 Advance is an advance developed technology of OCT which includes a system that provides a non-invasive method for obtaining 3D tomographic images of the posterior pole (ONH and retinal vasculature). The OCT-A allows scans at various retinal layers and provides the vessel density map and an automated measurement of the vascular density in the scanned region. We obtained the following measurements: circumpapillary vessel density of the whole image (wcpVD), percentage of vessel density at the optic nerve head (iVD) and macular capillary density of the whole image (wmVD). For this study, the imaging of scanning over a 4.5×4.5 mm^2^ areas centred on the optic disc and on the foveal centre was used. The measurement of vascular density in the external retinal nerve fiber layer (RNFL) layer, from the internal limiting membrane to the inner plexiform layer/inner nuclear layer +8 μm was considered.

The values of vessel density were quantified blindly and independently of the examiner since the value is automatically provided by the device.

### ONH colorimetric assessment

Photographs of each eye fundus were obtained using the non-mydriatic retinal camera Canon CR-DGi (Canon, Tokyo, Japan). ONH colorimetric evaluation determined the ONH Hb amount using the Laguna ONhE program, as described in previous studies.^15–^ ^20^ The percentages of the amount of ONH Hb were quantified blindly and independently of the examiner since the retinographies were sent to a third blind evaluator for the diagnosis of the patient who analysed the ONH images. This software uses algorithms to divide the optic disc into sectors and automatically identify the central ONH vessels by means of artificial intelligence systems for deep learning using convolutional neural networks.^21^ Two different zones were differentiated, those corresponding to the central ONH vessels (artery and vein) and ONH tissue itself. In order to be properly analysed and processed, retinographies have to meet a number of technical requirements since a certain alteration of the colours originated by the photograph is to be expected, such as the intensity of light or the focus. Therefore, blue, green and red levels in the image were assessed pixel by pixel using an image processing program based on MATLAB (MathWorks, Natick, MA, USA). The images that could be analysed with Laguna ONhE were those with sufficient luminosity to provide a good visualisation and focus of the optic papilla and the ONH and its vessels without having so much light as to be saturated in red or too dark as to lack blue. The influence of the lens was compensated for by analysing the differences between the green and blue components before calculating the results for the Hb amount. All images that did not pass the quality control of the Laguna ONhE program or images of poor quality due to media opacity or inadequate focus were excluded. The Laguna ONhE program examines three spectral components of ONH photographs: blue (B), green (G) and red (R). The equation (R–G)/R produces a value of G relative to R, which is used as the reference of the maximum Hb amount. The Hb levels present in the different tissue zones are calculated using this equation and expressed as a percentage relative to this maximum value. ONH areas with a high Hb content would reflect proportionally less green light. In contrast, areas with low Hb levels would reflect higher proportions of green as it is absorbed mainly by the blood.^15 17^

The analysis of six sectors of the ONH described previously^18^ was used to obtain the GDF index and estimates of the rim area and vertical cup-to-disc (C/D) ratio. In addition, we calculated the relative amount of Hb in the cup and in the six sectors of the rim. The distribution of the sectors at the neuroretinal rim was as follows: (1) nasal sector: from 121° to 230°; (2) nasal-inferior sector: from 270° to 231°; (3) temporal-inferior sector: from 271° to 310°; (4) temporal sector: from 311° to 40°; (5) temporal-superior sector: from 41° to 80°; (6) nasal-superior sector: from 81° to 120°.

Tissue Hb levels diminish from the disc periphery towards the centre and, accordingly, colours change proportionally such that Hb levels can be topographically determined.

### Statistical analysis

The Kolmogorov–Smirnov test was used to confirm the normal distribution of the quantitative data. After checking for a normal distribution of the variables data were compared between glaucomatous and normal subjects using Student’s t-test. Data are presented as mean±SD. Relationships between parameters were examined using Pearson correlation. Statistical comparisons between correlations were analysed using Cohen’s Q Method. Areas under receiver operating characteristic (ROC) curves (AUROCs) were calculated for all parameters determined in each test. To compare the AUROCs obtained with the different devices, the Hanley/McNeil method was used.^22^ All statistical tests were performed using the software package IBM SPSS (version 21.0; IBM Corp., Somers, NY, USA). Significance was set at p<0.05.

## RESULTS

A total of 112 eyes were included in this study. Of these eyes, three were excluded due to poor OCT-A images quality and another one due to a blurred retinography so it could not be analysed with the Laguna ONhE program. Therefore, data from 108 eyes were analysed. Table 1 summarises the demographic, ocular and clinical characteristics in the OAG and normal groups. There were no statistically significant differences in age, gender, diabetes mellitus, hypercholesterolaemia, arterial hypertension or disc area between glaucoma patients and control subjects (table 1).

**Table 1:** Demographic, clinical and ocular characteristics and comparison of vessel density (%) evaluated by OCT-A and amount of ONH Hb (%) determined by Laguna ONhE program between glaucoma and normal groups

	Normal (n=41)	Glaucoma (n=67)	P value
Gender (M/F)	9/32	27/40	0.05*
Age (years)	59.20±10.73	67.42±10.35	0.511†
Glaucoma medications	–	54 (80.6%)	–
Glaucoma surgery	–	11 (16.4%)	–
MD (dB)	–	4.57±2.03	–
HTA (yes/no)	9/32	15/52	0.959*
DM (yes/no)	9/32	11/56	0.473*
Hypercholesterolemy (yes/no)	12/29	11/56	0.113*
IOP (mm Hg)	14.54±1.77	15.12±2.05	0.136†
Disc area (mm^2^)	2.12±0.34	2.22±0.36	0.242†
Vertical C/D	0.42±0.13	0.72±0.11	<0.0001†
Horizontal C/D	0.49±0.15	0.76±0.13	<0.0001†
Laguna ONhE			
GDF	7.98±14.09	−18.76±13.31	<0.001†
Total Hb (%)	71.10±1.67	68.86±2.46	<0.001†
Cup Hb (%)	68.14±5.25	64.77±5.08	0.001†
Hb 311–40° (T) (%)	63.84±3.59	65.08±2.76	0.046†
Hb 41–80° (%)	72.43±2.26	70.64±3.33	0.003†
Hb 81–120° (%)	78.50±2.89	75.99±3.71	<0.001†
Hb 121–230º (%)	76.97±2.99	75.17±2.43	0.001†
Hb 231–270° (%)	77.64±2.74	75.78±3.49	0.004†
Hb 271–310° (%)	70.75±2.32	68.82±3.72	0.003†
Vessel density			
wcpVD (%)	22.18±3.42	16.03±2.89	<0.001†
iVD (%)	18.31±5.56	12.52±4.67	<0.001†
wmVD (%)	15.60±2.34	13.34±2.32	<0.001†

*χ^2^.

†Student’s t-test.

All above measurements are represented by mean±SD.

C/D, cup to disc ratio; cup Hb, amount of Hb at the optic nerve cup; DM, diabetes mellitus; GDF, glaucoma discriminant function; HTA, arterial hypertension; iVD, papilar vessel density; MD, mean defect; total Hb, total amount of Hb at the ONH; wcpVD, circumpapillary vessel density; wmVD: macular vessel density.

As depicted in table 1, 11 patients had undergone antiglaucomatous filtering surgery due to poor IOP control despite glaucoma medication.

Regarding the number of glaucoma treatment, 17 patients were on one eye drop, 30 on two and 7 on three ocular hypotensive medications.

The results on vessel density are shown in table 1. Significant differences between normal subjects and glaucoma patients in the wcpVD (22.18±3.42 vs 16.03±2.89%; p<0.001), iVD (18.31±5.56 vs 12.52±4.67%; p<0.001) and wmVD (15.60±2.34 vs 13.34±2.32%; p<0.001) were found.

In the same way, healthy subjects presented significantly more amount of papillary haemoglobin than glaucomatous patients (71.10±1.67 vs 68.86±2.46%; p<0.001), with significant differences in all papillary sectors except the temporal sector and in the papillary cup (68.14±5.25 vs 64.77±5.08%; p=0.001). The GDF index presented a negative value in the OAG patients and had positive values in healthy subjects (−18.76±13.31 vs 7.98±14.09; p<0.001) (table 1).

**Figure 2:**
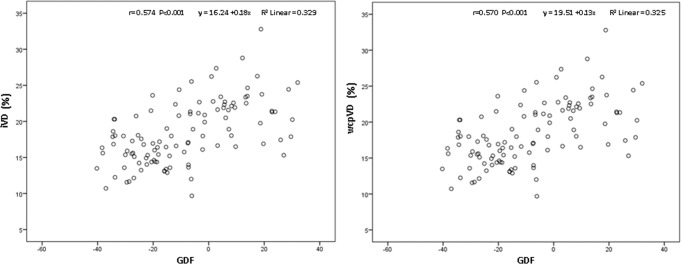
Correlation between the GDF index of Laguna ONhE and the circumpapillary vessel density, Pearson’s correlation coefficient 0.570, p<0.001(right) and the papilar vessel density, Pearson’s correlation coefficient 0.574, p<0.001(left).

**Figure 3:**
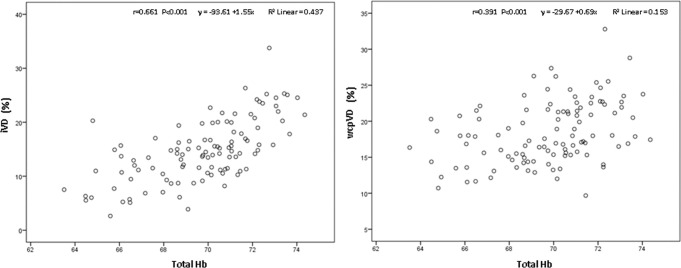
Correlation between the amount of Hb at the ONH and the circumpapillary vessel density, Pearson’s correlation coefficient 0.391, p<0.001 (right) and the papilar vessel density, Pearson’s correlation coefficient 0.661, p<0.001 (left).

Significant moderate correlation between peripapilar and papilar vessel density and Laguna ONhE GDF (r=0.570; p<0.001 and 0.574; p<0.001), figure 2, ONH Hb (r=0.391; p<0.001 and r=0.661; P<0.001), figure 3 and amount of Hb at the cup (r=0.243; p<0.001 and r=0.558; p<0.001) was found. Correlation between ONH Hb sectors and papilar vessel density was significant except in the temporal one, while correlation with peripapillary vessel density was significant in the upper and lower temporal and upper nasal sectors (table 2).

**Table 2:** Correlation between the Laguna ONhE program indices and the vessel density measured by OCT angiography

	wcpVD	iVD	wmVD
GDF	0.570(<0.001)*, ‡	0.574(<0.001)*, ‡	0.397(<0.001)*, ‡
Total Hb	0.391(<0.001)*, ‡	0.661(<0.001)*, ‡	0.235(0.109)‡
Cup Hb	0.243(0.001)*, ‡	0.558(<0.001)*, ‡	0.112(0.249)‡
Hb 311–40° (T)	−0.077(0.429)	0.086(0.379)‡	−0.112(0.250)‡
Hb 41–80°	0.355(<0.001)* ,‡	0.485(<0.001)*, ‡	0.121(0.211)‡
Hb 81–120°	0.268(0.005)*	0.344(<0.001)*	**0.194(0.044)†**
Hb 121–230°	0.141(0.145)	0.270(0.005)*	0.162(0.094)
Hb 231–270°	0.146(0.132)	**0.223(0.020)†**	**0.204(0.034)†**
Hb 271–310°	0.277(0.004)*	0.345(<0.001)*, ‡	0.150(0.120)‡

*Correlation at a significance level below p=0.01.

†Correlation at a significance level below p=0.05.

‡Comparison between correlations at a significance level below p=0.05 (Q-Cohen’s Method).

Statistical significant correlations are shown in bold values.

cup Hb, amount of Hb at the optic nerve cup; GDF, glaucoma discriminant function; iVD, papilar vessel density; total Hb, total amount of Hb at the ONH; wcpVD, circumpapillary vessel density.

ROC curves for all the variables determined using each diagnostic procedure and the cut-off providing sensitivity for 95% and 80% specificity determined in each case are shown in table 3.

**Table 3:** Areas under receiver operating characteristic curves (AUROCs) for the variables measured using the Laguna ONhE program and OCT-A

	AUROC (95% CI)	P value	Sensitivity at 95% specificity				Sensitivity at 80% specificity			
			Cut-off	Sensitivity (95% CI)	+LR	−LR	Cut-off	Sensitivity (95% CI)	+LR	−LR
Vessel density										
wcpVD	0.93(0.86 to 0.97)	<0.0001	≤17.3	73% (61–83)	14.99	0.28	≤19.9	88% (78–95)	4.51	0.15
wmVD	0.77(0.68 to 0.84)	<0.0001	≤11.6	22% (13–34)	4.59	0.82	≤13.8	60% (47–72)	3.06	0.50
iVD	0.79(0.70 to 0.86)	<0.0001	≤10.4	31% (21–44)	6.43	0.72	≤13.6	55% (43–67)	2.83	0.56
Laguna ONhE										
GDF	0.92(0.86 to 0.97)	<0.0001	≤-12.0	75% (63–85)	15.30	0.27	≤-3.7	88% (78–95)	4.51	0.15
Total Hb	0.78(0.69 to 0.85)	<0.0001	≤68.6	42% (30–55)	8.57	0.61	≤69.8	61% (49–73)	3.14	0.48
Cup Hb	0.70(0.60 to 0.78)	0.0002	≤57.3	7% (3–17)	1.53	0.97	≤63.8	45% (33–57)	2.29	0.69
Hb 121–230°	0.67(0.57 to 0.75)	0.0027	≤72.7	16% (8–28)	3.37	0.88	≤74.3	36% (25–49)	1.84	0.80
Hb 231–270°	0.67(0.57 to 0.76)	0.0012	≤73.3	24% (14–36)	4.90	0.80	≤74.9	43% (31–56)	2.22	0.70
Hb 271–310°	0.67 (0.57 to 0.76)	0.0010	≤67.3	25% (16–38)	5.20	0.78	≤69.5	55% (43–67)	2.83	0.56
Hb 311–40° (T)	0.62 (0.52 to 0.71)	0.0432	>69.9	3% (0.4–10)	0.61	1.02	>66.4	28.4% (18–41)	1.45	0.89
Hb 41–80°	0.69 (0.59 to 0.77)	0.0003	≤68.6	24 (14–36)	4.90	0.80	≤70.8	54% (41–66)	2.75	0.57
Hb 81–120°	0.72 (0.63 to 0.81)	<0.0001	≤75.1	43 (31–56)	8.87	0.60	≤76.4	60% (47–72)	3.06	0.50

AUROC, area under the ROC curve; cup Hb, amount of Hb at the optic nerve cup; GDF, glaucoma discriminant function; iVD, papilar vessel density; LR, likelihood ratio; total Hb, total amount of Hb at the ONH; wcpVD, circumpapillary vessel density; wmVD, macular vessel density.

Overall, the AUROC for discriminating between healthy and glaucomatous eyes were higher for the OCT-A wcpVD and the Laguna ONhE GDF index, 0.93; 95% CI 0.86 to 0.97, p<0.0001 vs 0.92; 95% CI 0.86 to 0.97, p<0.0001. The OCT-A iVD and Laguna ONhE ONH Hb parameters showed lower AUROCs, 0.79; 95% CI 0.70 to 0.86; p<0.0001 vs 0.78; 95% CI 0.69 to 0.85, p<0.0001, respectively.

GDF had the greatest sensitivities with a specificity of 95% (wcpVD had a sensitivity of 73%, a positive likelihood ratio of 14.99 and a negative likelihood ratio of 0.28 and GDF had a sensitive of 75%, a positive likelihood ratio of 15.30 and a negative likelihood ratio of 0.27) and a specificity of 80% (wcpVD had a sensitivity of 88%, a positive likelihood ratio of 4.51 and a negative likelihood ratio of 0.15 and GDF had a sensitive of 88%, a positive likelihood ratio of 4.51 and a negative likelihood ratio of 0.15).

**Figure 4:**
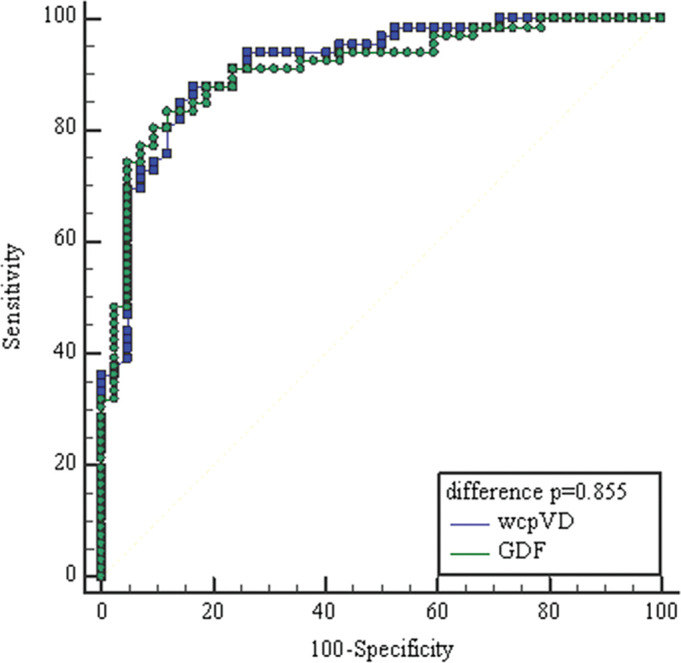
Receiver operating characteristic curves of best parameters of OCT angiography and Laguna ONhE. The area under the curve (95% CI) for wcpVD index, peripapilar vessel density, (blue squares line) was 0.93 (0.86 to 0.97) and GDF index, glaucoma discriminant function, (green circles line) 0.92 (0.86 to 0.97).

Pairwise comparisons showed that the AUROC of wcpVD (0.93) was not significantly different than GDF (0.92) (p=0.855), and their diagnostic accuracies were similar for differentiating between glaucoma and healthy eyes (figure 4).

## DISCUSSION

Optic nerve perfusion depends on three factors, namely, oxygen saturation, blood flow and Hb content. It has been suggested that changes in ONH reflectance can detect variations in Hb levels, which may be useful for indirectly measuring ONH perfusion.^15 23^

Optic nerve ischemia and reduced ocular blood flow have been shown to be associated with the development and progression of glaucoma.^24–^ ^26^

The OCT-A Nidek RS-3000 Advance allows the quantification of vascular areas and vessel density, and it is capable of identifying changes in the papilar, circumpapilar and macular vessel density in glaucoma patients. Decreased vessel density has been associated with the severity of visual field damage independently of the structural loss.^9^ Its use in the clinic is increasingly widespread and its diagnostic yield in glaucoma has been demonstrated.^27 28^

The results obtained with OCT-A showed higher papilar, circumpapilar and macular vessel density in the control group than in glaucoma patients. This is consistent with previous studies conducted with OCT-A that support the value of vascular compromise in glaucoma patients.^29–^ ^32^

On the other hand, it is known that glaucoma patients have paler papillary colouration. Papillary pallor is determined by lower amount of haemoglobin associated with lower tissue perfusion. In this study, Laguna ONhE software was used to analyse the colour changes in the ONH using Hb as a reference pigment, compensating for different variables such as illumination or lens absorption and diffusion.

The purpose of this program is not only to measure the perfusion of the ONH, but also to detect the paleness caused by a decrease in capillary density and, consequently, an axonal loss.^15^ It has been shown that Laguna ONhE software has high accuracy and reproducibility in early diagnosis of patients with incipient glaucoma compared with the traditional functional and structural tests used for this purpose.^15–^ ^20^

The colorimetric assessment of the glaucomatous ONH carried out through Laguna ONhE program has identified greater pallor in glaucomatous patients, both globally, which implies the whole papilla, and in the area of the ONH cup.

Significant differences in all papillary sectors were found between glaucoma patients and normal subjects except the temporal sector. The temporal sector described by Laguna ONhE comprises from 311° to 40°, that is, the temporal horizontal area.^18^ Typically, superotemporal and inferotemporal sectors are considered as specific to glaucoma and the temporal sector is less discriminatory.^33^ In superotemporal (41–80°) and inferotemporal (271–310°) sectors significant differences between the two groups were found.

Pena Betancor *et al* ^18^ calculated the relative amount of Hb in sectors of the ONH from stereoscopic colour fundus images using the Laguna ONhE method in 87 healthy eyes and 71 glaucomatous eyes. The MD values were higher than those found in our glaucoma patients. Our results agree with those found by Pena Betancor *et al.* Relative Hb amount presented significant reduction in all rim sectors, except in the temporal 311° to 40°. It is possible that the amount of Hb in the temporal sector could be the parameter with the least difference between normal subjects and glaucoma patients and might be related to the smaller size of the neuroretinal rim.

GDF parameter of Laguna ONhE program combines the results of the amount of Hb obtained in various regions of the ONH to differentiate between glaucoma and normality. This combination might improve the diagnostic performance of ONH Hb analysis alone.

Moreover, GDF parameter of Laguna ONhE has revealed to have a high diagnostic yield similar to that of the wcpVD or circumpapilar vessel density of the OCT-A, which has shown to have a higher diagnostic yield than the papilar or macular vessel density measurements in previous studies.^27 31 32^ Of these two parameters, GDF shows better levels of sensitivity as well as positive and negative likelihood ratio when a specificity of 80% is pre-established.

It seems, in view of our results, that both methods are capable of identifying changes in glaucoma patients, related to altered ONH microcirculation. Furthermore, the most relevant parameters of the OCT-A and the Laguna ONhE program are moderately correlated, which might suggest that both measurement methods, although quantifying glaucomatous damage in different ways, might measure changes at the level of papillary microcirculation in glaucoma patients.

The correlation between the amount of ONH Hb and papillary vessel density was greater than that found with circumpapillary vessel density. This makes sense since ONH colouring depends on the amount of Hb contained in the papilla and therefore is more related to the amount of vessels present at the papilla than circumpapillary. The GDF index correlates significantly with papillary, circumpapillary and macular vessel density, and patients with lower vessel density showed more negative or pathological GDF values, as we have already described in previous studies.

It is unclear whether vascular changes are a primary or secondary consequence in glaucoma. Reduced retinal perfusion leads to faster retinal ganglion cell death, but on the other hand, OCT-A may detect lower metabolic demands or vascular dropout due to retinal ganglion cell dysfunction. Moghimi *et al* ^11^ found that OCT-A parameters were significantly associated with RNFL decline rate in patients with mild to moderate POAG followed up over time. Patients with lower circumpapilar and macular vessel density baseline tended to progress significantly faster than those with higher values.

Yarmohammadi *et al* ^9^ demonstrated the ability of OCT-A to discriminate between glaucoma suspects and healthy subjects. It should be noted that patients included in our study presented a MD under 6 dB, being patients with incipient glaucoma and that mean ONH cup was 0.7, which indicates that both Laguna ONhE and OCT-A can detect changes related to microcirculation even in early stages of the disease.

In view of the results and in the absence of studies with a greater number of patients, it seems that both procedures, although quantifying glaucomatous damage in different ways, can identify changes in papillary microcirculation in patients with glaucoma and have the same diagnostic validity.

The colorimetric analysis procedure offers some advantages over OCT-A. The first is related to the length of the OCT-A examination. One of the disadvantages of OCT-A is the duration of the exam, which is longer than the scanning time with SD-OCT, which may imply a limitation when exploring uncooperative patients. In this study, four patients could not be included in the final data analysis, three of them due to lack of collaboration that made it impossible to complete the analysis with OCT-A. Poor OCT-A image quality due to segmentation errors made OCT-A unreadable. However, Laguna ONhE program can be used in patients that either because of their low visual acuity or their age are not able to maintain their sight in a pre-established fixation point, since the analysis of the amount of ONH Hb only requires a good quality retinography, even if the image of the papilla is off-centre, as we have already demonstrated in previous studies carried out on patients with greater difficulties such as those suffering from childhood glaucoma. In fact, an interesting application of the method could be the diagnosis and follow-up of these patients.^34^

An additional advantage of Laguna ONhE over OCT-A is its price, which is determined by the cost of the fundus camera, a fundamental device in any ophthalmological clinic. Laguna ONhE is a non-invasive method that is not expensive since it is performed on retinographies. Diagnostic devices such as OCT-A are inaccessible to many ophthalmologists.

Our study has limitations. Not all healthy subjects were evaluated with visual field since most of them were healthy volunteers who came to the ophthalmological consultations for refraction or annual check-ups. However, all the subjects included in the study had a normal nerve fibre layer analysis, and all the healthy participants were found to be normal. Another limitation is the sample size as this was a preliminary study, carried out with an OCT-A which was lent during a limited period of time in order to carry out research studies. Despite the small sample size, enough statistical power, above 0.80, was achieved in the comparisons of parameters.

In conclusion, the amount of haemoglobin and peripapilar, papilar and macular vessel density are well correlated and present lower values in eyes of glaucoma patients. Both methods have similar diagnostic validity in open-angle glaucoma patients.
